# Evidence to support a drain-free strategy in kidney transplantation using a retrospective comparison of 500 consecutively transplanted cases at a single center

**DOI:** 10.1186/s12893-021-01081-x

**Published:** 2021-02-05

**Authors:** Ahmed Farag, Jeffrey J. Gaynor, Giuseppe Serena, Gaetano Ciancio

**Affiliations:** 1 Department of Surgery, University of Miami Miller School of Medicine, Miami, FL USA; 2 Department of Urology, University of Miami Miller School of Medicine, Miami, FL USA; 3 Miami Transplant Institute, University of Miami Miller School of Medicine, Miami, FL USA; 4 Department of Surgery, Zagazig University School of Medicine, Zagazig, Egypt; 5 Department of Surgery, Nassau University Medical Center, East Meadow, NY USA; 6 Department of Surgery and Urology, University of Miami Miller School of Medicine, Jackson Memorial Hospital, Miami, FL USA; 7 Miami Transplant Institute, 1801 NW 9th Ave, 7th Floor, Miami, FL 33136 USA

**Keywords:** Kidney transplant, Drain-free, Collection, Lymphocele, Wound

## Abstract

**Introduction:**

Routine placement of surgical drains at the time of kidney transplant has been debated in terms of its prognostic value.

**Objectives:**

To determine whether the placement of a surgical drain affects the incidence rate of developing wound complications and other clinical outcomes, particularly after controlling for other prognostic factors.

**Methods:**

Retrospective analysis of 500 consecutive renal transplant cases who did not (Drain-free, DF) vs. did (Drain, D) receive a drain at the time of transplant was performed. The primary outcome was the development of any wound complication (superficial or deep) during the first 12 months post-transplant. Secondary outcomes included the development of superficial wound complications, deep wound complications, DGF, and graft loss during the first 12 months post-transplant.

**Results:**

388 and 112 recipients had DF/D, respectively. DF-recipients were significantly more likely to be younger, not have pre-transplant diabetes, receive a living donor kidney, receive a kidney-alone transplant, have a shorter duration of dialysis, shorter mean cold-ischemia-time, and greater pre-transplant use of anticoagulants/antiplatelets. Wound complications were 4.6% (18/388) vs. 5.4% (6/112) in DF vs. D groups, respectively (P = 0.75). Superficial wound complications were observed in 0.8% (3/388) vs. 0.0% (0/112) in DF vs. D groups, respectively (P = 0.35). Deep wound complications were observed in 4.1% (16/388) vs. 5.4% ((6/112) in DF vs. D groups, respectively (P = 0.57). Higher recipient body mass index and ≥ 1 year of pre-transplant dialysis were associated in multivariable analysis with an increased incidence of wound complications. Once the prognostic influence of these 2 factors were controlled, there was still no notable effect of drain use (yes/no). The lack of prognostic effect of drain use was similarly observed for the other clinical outcomes.

**Conclusions:**

In a relatively large cohort of renal transplant recipients, routine surgical drain use appears to offer no distinct prognostic advantage**.**

## Introduction

Advances in immunosuppressive protocols and refinements in surgical techniques have been accompanied by decreased morbidity and improved patient and graft survival in renal transplant recipients. However, surgical complications may still occur, which increases post-operative morbidity with longer hospital stay, more frequent follow-up, higher readmission rates, and a slower recovery [1]. Wound complications as a subcategory are the most common type of transplant-associated surgical complications, but fortunately, their occurrence has not been associated with any decreases in graft or patient survival [2]. Wound complications can be divided into superficial (at or above the fascia) and deep (below the fascia). Renal transplant recipients are at an increased risk for wound complications owing to a number of (transplant recipient-related and surgical technique-related) risk factors associated with slower wound healing [3]. For instance, obesity, older age, pre-transplant malnutrition, pre-transplant diabetes mellitus, prolonged pre-transplant dialysis, pre-transplant uremia, the occurrence of delayed graft function (DGF), and post-transplant use of specific immunosuppressive agents such as mTOR inhibitors are transplant-related risk factors that may affect wound healing following renal transplantation, in addition to surgical technique-related risk factors (e.g., extensive dissection, excessive use of electrocautery, and poor hemostasis) [4–7]. While different immunosuppression protocols may affect the rates of developing wound complications and lymphoceles in renal transplant recipients [8–11], the surgeon’s experience has also been reported to affect the rate of developing wound complications [5].

Using an intraoperative drain during renal transplantation in order to decompress the surgical site, with the goals of (i) achieving more rapid monitoring of any intra-abdominal bleeding and urine leaks that may occur, as well as (ii) reducing the incidence and severity of wound complications, has been a matter of debate. Historically, according to Halstead and Howard Kelly, “The more imperfect the technique of the surgeon the greater the necessity for drainage,” and “Drainage is a confession of imperfect surgery,” respectively [12–14]. Conversely, the drain may be a conduit for easy access of pathogens and put the immunosuppressed renal transplant recipient at an increased risk for developing wound complications including infection [15–21]. There are some published retrospective studies that analyzed the placement of a prophylactic drain in renal transplant recipients and concluded that a clear favorable effect of drain use in reducing the incidence of wound complications was not found, although the incidence of perirenal collections appeared to be reduced when using a surgical drain [22–25]. While routine placement of a surgical drain at the time of kidney transplant had been performed at our center prior to 2014, since then the thinking about its necessity to even be used had changed. The aim of this study was therefore to determine in a relatively large retrospective review of 500 consecutively transplanted cases whether intraoperative surgical drain placement at the time of renal transplantation, in fact, has any role in preventing the development of wound complications post-transplant.

## Patients and methods

After obtaining Institutional Review Board approval, written informed consent from all study participants, and following the ethical principles (as revised in 2013) of the Helsinki Declaration [26], a retrospective analysis of 500 consecutive kidney (± other organ) transplants performed at the Miami Transplant Institute between January 2014 and September 2019 was performed; all patients were followed for a minimum of 12 months post-transplant. This group of recipients represented a single surgeon’s experience in a program where previously, the surgical drain had been routinely used.

All renal allografts were placed in the iliac fossa, and the recipient´s external iliac vessels were dissected free, with limited dissection to the anterior wall (not the whole circumference) of the external iliac vessels. After completing the vascular anastomoses, a modified extravesical ureteroneocystostomy technique was used in all recipients [27]. Based on intraoperative placement of a surgical (Jackson Pratt, JP) drain during the transplant (yes/no), recipients were retrospectively divided into 2 groups; drain-free (DF) and drain (D) groups. Discretion to use a surgical drain was merely random, i.e., the decision to place/not place a drain was not based on any specific risk factor model for the likelihood of developing a wound complication. In the D group, < 50 mL drainage over 24 h was an indication for drain removal.

All patients received dual induction immunosuppression with rabbit antithymocyte globulin/basiliximab and maintenance consisting of tacrolimus/mycophenolic acid and corticosteroid avoidance [28–30]. Pre-operative single prophylactic dose of a 1st generation cephalosporin (or quinolone in case of a reported allergy to the former) was given in the attempt to prevent the development of a post-operative wound infection.

Baseline variables such as group status (DF vs. D), recipient age, gender, race and ethnicity, body mass index, history of pre-transplant diabetes, pre-transplant duration of dialysis and dialysis status, pre-transplant use of anticoagulants and antiplatelets, donor type, and number of donor renal allograft arteries were analyzed for their associations with various clinical outcome variables. Again, it was of primary interest to determine the prognostic value of DF vs. D.

The primary outcome was the occurrence of a wound complication (yes/no), whether superficial (at or above the fascia) or deep (below the fascia) during the first 12 months post-transplant. Superficial wound complications (secondary outcome) included subcutaneous seroma, wound infection, and wound dehiscence without fascia disruption, while deep complications (secondary outcome), whether perirenal or pelvic, included hematomas, collections, and lymphoceles. A collection was defined as a perirenal or pelvic seroma, while lymphocele was defined as a cystic collection containing lymph fluid that was encapsulated with fibrous tissue adjacent to the graft [31, 32]. A deep abscess was defined as a wound infection if an isolated microorganism was identified in addition to clinical symptoms (pain at the graft site and fever). Once a deep wound complication was clinically suspected, ultrasound and computed tomography imaging studies were utilized.

Observation and frequent dressings were used in resolving a subcutaneous seroma and wound dehiscence, respectively, without the need for further intervention. Image-guided drainage was used if a deep complication failed to resolve conservatively. In most of the hematoma cases, surgical treatment was the definitive therapeutic option.

### Statistics

Frequency distributions were determined for baseline categorical variables, and the mean along with standard error (± SE) were calculated for baseline continuous variables. The primary outcome variable for this study was the development of a wound complication during the first 12 months post-transplant. Secondary outcome variables included the development of superficial (i.e., subcutaneous seroma or wound dehiscence) and deep wound complications (i.e., lymphocele, perirenal collection, or hematoma), as well as the development of DGF, and graft loss (death-censored and death-uncensored) during the first 12 months post-transplant. Graft loss was defined as the date of graft failure (return to permanent dialysis or graft nephrectomy) or death, whichever occurred first. Tests of association between baseline variables and the various time-to-event outcomes were performed using the log-rank test. Multivariable analysis was performed using stepwise logistic, Cox, and linear regression. Tests of association between baseline variables were performed using Pearson (uncorrected) chi-squared tests. P-values < 0.05 were considered to be statistically significant.

Stepwise logistic regression to determine the significant multivariable predictors of the likelihood of receiving a JP drain (yes/no) was also performed along with resulting propensity scores [33]. Propensity scores are typically used as a way to control for the effects of any unbalanced distributions of other potentially important baseline prognosticators existing between two study groups. By controlling for these imbalances (i.e., selection bias) in the statistical analysis, the “adjustment for propensity scores” approach attempts to ensure that an unbiased comparability exists between the 2 study groups (in this case, receiving vs. not receiving a JP drain). Here, the final Cox model for the hazard rate of developing a wound complication during the first 12 months post-transplant (primary outcome) was re-run after controlling for the propensity of receiving a JP drain.

## Results

Of 500 kidney transplants included in this study, 77.6% (388/500) had no drain (DF group) and 22.4% (112/500) had an intraoperative drain placed (D group). Recipients, who received a kidney alone transplant constituted 98.2% (381/388) of the DF group, and 81.2% (91/112) of the D group. In the DF group, 1.5% (6/388) and 0.3% (1/388) of the recipients received kidney/liver and kidney/intestine transplants, respectively. In the D group, 15.2% (17/112), 1.8% (2/112) and 1.8% (2/112) of the recipients received kidney/liver, kidney/intestine and kidney/heart transplants, respectively.

All patients who were alive with a functioning graft as of the last follow-up date, October 1, 2020, had a minimum follow-up of at least 12 months post-transplant. Mean recipient age was 50.7 (± 0.8) years. There were no significant associations between group status (DF vs D) and other baseline variables such as recipient gender, race and ethnicity, body mass index, presence of two or more donor renal arteries, and patients who received re-transplants. However, there were significant differences (Table 1) between the groups in mean age (DF < D, P = 0.00005), having pre-transplant diabetes mellitus (DF < D, P = 0.0005), pre-transplant use of anticoagulants or antiplatelets (DF > D, P = 0.001), pre-transplant duration on dialysis (DF < D, P = 0.00006), deceased donor recipient (DF < D, P < 0.000001), mean cold ischemia time (DF < D, P = 0.00001), and kidney transplant combined with other organs (DF < D, P < 0.000001).

**Table 1:** Associations of selected baseline variables with JP drain use (No/Yes)

Baseline variable	Use of JP drain		P-value
	No (N = 388)	Yes (N = 112)	
Mean recipient age (yr)	49.2 ± 0.9 (N = 388)	55.7 ± 1.3 (N = 112)	0.00005
Recipient age ≥ 50 yr	58.2% (226/388)	75.0% (84/112)	0.001
Male recipient	62.6% (243/388)	64.3% (72/112)	0.75
Black (non-Hispanic) recipient	35.8% (139/388)	34.8% (39/112)	0.85
Hispanic recipient	35.1% (136/388)	34.8% (39/112)	0.96
Mean recipient BMI (kg/m^2^)	26.4 ± 0.3 (N = 388)	27.5 ± 0.5 (N = 112)	0.07
Recipient BMI ≥ 25 (kg/m^2^)	57.5% (223/388)	67.0% (75/112)	0.07
Recipient pre-transplant DM	26.5% (103/388)	43.8% (49/112)	0.0005
On any anticoagulants or antiplatelets pre-transplant	61.1% (237/388)	43.8% (49/112)	0.001
Received preemptive transplant	22.4% (87/388)	12.5% (14/112)	0.02
Pre-transplant time on dialysis (mo)	36.9 ± 1.9 (N = 388)	58.4 ± 4.8 (N = 112)	0.00006
Pre-transplant time on dialysis ≥ 12 mo	66.0% (256/388)	76.8% (86/112)	0.03
Donor age (yr)	40.5 ± 0.7 (N = 388)	43.6 ± 1.3 (N = 112)	0.04
Donor age ≥ 50 yr	31.7% (123/388)	41.1% (46/112)	0.06
Number of donor arteries ≥ 2	26.3% (102/388)	25.0% (28/112)	0.78
DD kidney recipient	58.2% (226/388)	86.6% (97/112)	< 0.000001
Mean CIT (hr)	16.9 ± 0.8 (N = 388)	23.5 ± 1.2 (N = 112)	0.00001
CIT ≥ 18 hr	51.0% (198/388)	70.5% (79/112)	0.0003
Kidney plus other organs transplanted	1.8% (7/388)	18.8% (21/112)	< 0.000001
Retransplanted kidney	8.0% (31/388)	7.1% (8/112)	0.77
Transplanted during 2018–2019	41.8% (162/388)	11.6% (13/112)	< 0.000001

*JP* Jackson Pratt, *BMI* Body Mass Index, *yr* year, *DM* diabetes mellitus, *mo* month, *hr* hour, *DD* deceased donor, *CIT* cold ischemia time

Four baseline characteristics were selected into the logistic regression model to predict a greater likelihood of receiving a JP drain (listed by order of selection): earlier date of transplant (continuous variable, P < 0.000001), received a multi-organ transplant (P < 0.000001), longer time (in months) on dialysis (continuous variable, P < 0.000001), and the recipient having pre-transplant diabetes mellitus (P = 0.000008). Once these 4 baseline variables were selected into the multivariable model, none of the other baseline variables had any further association with JP drain use (yes/no).

Although 4.6% (18/388) of DF and 5.4% (6/112) of D groups developed a wound complication (superficial or deep), this difference was clearly not significant (P = 0.75). In the DF group, 0.8% (3/388) developed a superficial wound complication, subcutaneous seroma (1/3) and wound dehiscence (2/3), while 0.0% (0/112) developed a superficial wound complication in the D group (P = 0.35). Times to occurrence were 0.4 (seroma) and 0.5–3.7 (wound dehiscence) months post-transplant.

In the DF group, 4.1% (16/388) developed a deep wound complication (lymphocele, perirenal collection, or transplant surgery-related hematoma), while in the D group, 5.4% (6/112) developed a deep wound complication (P = 0.57). Lymphocele was detected in 0.5% (2/388) in the DF group (at 2.4 and 9.1 months post-transplant) vs. 0.0% (0/112) in the D group (P = 0.44). The lymphoceles were managed conservatively. Perirenal collection was diagnosed in 11 patients overall, 1.8% (7/388) in DF vs. 3.6% (4/112) in D (P = 0.25), with the median time to occurrence being 0.9 (range: 0.5–6.1) months post-transplant. Perirenal collections were managed with image-guided drainage, with mean time to resolution being 1.71 (± 0.20) and 1.73 (± 0.50) weeks in 7 DF and 4 D patients during the first year post-transplant, respectively (P = 0.98). Hematomas occurred equally in both groups, 1.8% (7/388) in DF and 1.8% (2/112) in D (P = 0.99), with the median time to occurrence being 0.2 (range: 0.1–0.5) months post-transplant. Most hematomas were surgically evacuated (7/9), while watchful waiting was practiced in 2 cases. Association of DF vs. D groups with wound complications are shown in Table 2.

**Table 2:** Univariable associations of JP drain usage (no/yes) with selected outcomes variables during the first 12 months post-transplant (N = 500)

	Drain-free (N = 388)	Drain (N = 112)	P-value
Wound complications	4.6% (18/388)	5.4% (6/112)	0.75
*Superficial wound complications*	0.8% (3/388)	0.0% (0/112)	0.35
1. Subcutaneous seroma	0.3% (1/388)		
2. Wound dehiscence	0.5% (2/388)		
*Deep wound complications*	4.1% (16/388)	5.4% (6/112)	0.57
1. Perirenal collection	1.8% (7/388)	3.6% (4/112)	0.25
2. Hematoma	1.8% (7/388)	1.8% (2/112)	0.99
3. Lymphocele	0.5% (2/388)	0.0% (0/112)	0.44
Delayed graft function	12.4% (48/388)	17.9% (20/112)	0.14
Death-censored graft failure	1.5% (6/388)	3.6% (4/112)	0.20
Death with a functioning graft	2.8% (11/388)	2.7% (3/112)	0.90
Death-uncensored graft loss	4.4% (17/388)	6.3% (7/112)	0.47

In multivariable analysis, 2 baseline variables were associated with an increased incidence of developing wound complications during first 12 months post-transplant: higher body mass index (P = 0.00007) and a pre-transplant duration of at least one year on dialysis (P = 0.004). No association of drain use with the incidence of surgical site complications was observed after controlling for these 2 factors (P = 0.80). In addition, the multivariable test to include JP drain use after controlling for the propensity to receive a JP drain yielded P = 0.98—again, showing no significant impact of JP drain use (yes/no) on the hazard rate of developing a wound complication during the first 12 months post-transplant.

Numerically, among 283 patients having either a pretransplant body mass index < 25 kg/m^2^ or < 12 months of pre-transplant dialysis (i.e., patients identified at lower risk), the observed percentage of patients who developed a wound complication during the first 12 months post-transplant was 1.3% (3/232) vs. 2.0% (1/51) for the DF vs. D groups. Among 217 patients having a body mass index ≥ 25 kg/m^2^ and ≥ 12 months of pre-transplant dialysis (i.e., patients identified at increased risk), the observed percentage of patients who developed a wound complication during the first 12 months post-transplant was 9.6% (15/156) vs. 8.2% (5/61) for the DF vs. D groups. Thus, once the prognostic effects of pretransplant body mass index and time (in months) on dialysis were controlled, no notable association of JP drain use (yes/no) with the risk of developing a post-transplant wound complication was observed. Similar results were found when analyzing the hazard rate of developing a deep wound complication (results not shown).

DGF was non-significantly lower in DF (12.4%, 48/388) than D (17.9%, 20/112) (P = 0.14). In multivariable analysis, 4 risk factors (use of deceased donor allografts, higher recipient body mass index, pretransplant duration of dialysis ≥ 1 year, and male recipient) were associated with an increased incidence of DGF (Table 3); the use of drain did not affect DGF incidence in multivariable analysis (P = 0.65).

**Table 3:** Selected linear model (obtained via stepwise regression) for the likelihood of developing delayed graft function (68 events)

Variable	Multivariable model	
	P-value	Coeff ± SE
DD recipient	< 0.000001	0.175 ± 0.033
Higher recipient BMI	0.0005	0.0092 ± 0.0026
Time on dialysis ≥ 12mo	0.01	0.084 ± 0.034
Male recipient	0.01	0.074 ± 0.030

*DD* deceased donor, *BMI* body mass index, *mo* months, *Coeff* Coefficient, *SE* standard error

Lastly, death-censored graft failure, death with a functioning graft, and death-uncensored graft failure rates during the first 12 months post-transplant were not statistically different between the DF and D groups (1.5%, 6/388 vs. 3.6%, 4/112, P = 0.20 for death-censored graft failure; 2.8%, 11/388 vs. 2.7%, 3/112, P = 0.90 for death with a functioning graft; and 4.4%, 17/388 vs. 6.3%, 7/112, P = 0.47 for death-uncensored graft failure). Two patients (2/10 who developed graft failure) lost their grafts due to fracture of upper pole of the graft and bleeding, one in each group.

## Discussion

We are reporting here a single surgeon’s experience at a kidney transplant program in which the surgical drain had routinely been used prior to 2014. Given the fact that the previously reported kidney transplant studies comparing no drain use vs. drain use have not demonstrated any clear superiority of intraoperative drain placement in reducing the incidence rate of post-transplant wound complications, as well as the fact that intraoperative drain placement may actually provide a conduit for easy access of pathogens (thereby, potentially increasing the risk of wound complication development), the rationale to change the routine practice and introduce a drain-free strategy at our center began in 2014. The results of this retrospective study of 500 consecutively transplanted patients by a single surgeon between 2014 and 2019 clearly support the results reported by others in showing no clinical benefit of intraoperative drain placement in kidney transplantation.

Numerous studies have addressed the issue of using drains in surgical patients. In non-transplant abdominal surgical patients, the use of drains did not affect morbidity or mortality rates [17, 18, 20, 21]. Renal transplant recipients are at a higher risk for wound complications due to pre-transplant malnutrition and chronic anemia, impaired immunity resulting from the post-transplant use of immunosuppressive agents, and therefore experience decreased wound healing and repair, with an overall incidence rate of surgical complications of 15–32% [4, 34–37]. The role of drain placement in decreasing wound complication rates (specifically, for lymphoceles and perirenal collections) in renal transplant patients is debatable.

The overall observed incidence of wound complications in our study was 4.8% (24/500), being 4.6% (18/388) in DF and 5.4% (6/112) in D groups, which is much lower than previously reported rates. Placement of the drain at the time of transplant did not affect the rate of developing superficial wound complications, and the observed overall incidence of superficial wound complications in our study (0.6%, 3/500) was lower here than the reported rates of 5–8% [38]. For example, Sidebottom et al. reported higher rates of superficial wound complications of 8.5% and 12.2% in the DF and D groups, with no prognostic effect of drain placement (P = 0.2) [22]. Similarly, Cimen et al. [24] reported no significant prognostic effect of drain placement on the incidence of all wound complications despite a somewhat higher rate of wound complications in DF (23.7%) compared to D (14.2%) groups (p = 0.06). In our study, there was no set criteria used in determining which patients should (vs. should not) receive drain placement, while drain placement was intentionally used more often in patients with known risk factors for impaired wound healing in the previously mentioned studies.

Our study also showed that drain placement did not significantly affect the rate of developing a deep wound complication in either univariable or multivariable analysis. Sidebottom et al. [22] reported that the rates of deep wound complications in renal transplant recipients were 8.5% in both the DF and D groups, with hematomas being the most common organ-space surgical site complication in DF (5.5%) and D (6.7%) groups, with drain placement not affecting the incidence rate of developing a deep wound complication.

The overall incidence of perirenal collections was 2.2% (11/500) in our cohort, and the use/nonuse of drain did not significantly affect this outcome, being non-significantly higher in D (3.6%, 4/112) than DF (1.8%, 4/388) (P = 0.25). The reported incidence of perirenal collections was 19.8%/26% in Cimen et al. [24], 1.9%/3% in Sidebottom et al. [22], 16%/45.2% in Derweesh et al. [25], and 23.6%/30.6% in Atray et al. [23], in D (control)/DF patients. In contrast, Derweesh et al. [25] reported a significant effect of drain placement in reducing the rate of perirenal collections from 45.2% in DF to 16% in D groups. The previously mentioned study used mTOR inhibitors, and the reduction occurred in 58% of the patients who received mTOR inhibitors compared to 16.1% in the patients who received non-mTOR inhibitors [25]. Thus, impaired wound healing seen with mTOR inhibitors may force the surgeon to place a drain at the time of transplant [25, 37, 39, 40].

In this study, the rate of lymphocele development was quite low compared with the reported rates of 0.6–26% after renal transplantation [41–43], possibly owing to our having adopted a technique of limited external iliac vessels dissection resulting in less disturbance of lymphatics in the recipient. Cimen et al. [24] reported a statistically significant association between the presence of drain and having a higher risk of developing a perirenal collection (28%, P = 0.008), and they grouped perirenal seroma, collection and lymphocele into one category, “perirenal collection” [44, 45].

In our study, recipients with a higher body mass index and who had been receiving dialysis for one year or longer were at an increased risk for developing a wound complication, and drain placement was found to offer no specific advantage regarding the incidence rate of developing a wound complication.

DGF may affect the time-to-recovery from the pre-transplant long-term uremia causing chronic anemia, low albuminemia and interstitial edema; therefore, DGF may indirectly result in impaired wound healing [22, 46]. In this study, in either univariable or multivariable analysis, the absence of drain placement did not seem to be associated with an increased risk of developing DGF and consequently, did not affect the recovery time from the chronic anemia, hypoalbuminemia and edema that potentially affects the wound healing. Our results were consistent with Sidebottom et al. [22] who reported no association between the use of drain (Y/N) and the incidence of wound complications in renal transplant recipients.

There were several study limitations. The first and most important limitation is that this study was performed by a single surgeon at a single center; thus, these results may not be generalizable to those of other surgeons. While the Miami Transplant Institute is one of the largest kidney transplant centers, with 300–500 kidney transplants having been performed at our center per year since 2014, the clinical results for the patients transplanted by the other surgeons at our center were not available. A second limitation was that we had no set criteria for not using vs. using drain placement at the time of transplant in our patients, and the gold standard for comparing two groups such as DF vs. D in renal transplant patients would be to perform a randomized controlled trial. While the results of this study were consistent with those reported by others, suggesting (with the improvements made in kidney transplant surgical techniques over the years) that intraoperative drain placement is no longer necessary, the only way to convincingly answer this question would be to perform a randomized clinical trial. Conversely, while our study was retrospective in nature, the results were based on a rather large cohort. In addition, the fact that one surgeon performed 500 consecutive renal transplants at a single center may have helped in eliminating surgeon-to-surgeon differences in surgical dissection and technique; thus, potentially greater homogeneity in patient outcomes may have been observed within this cohort.

## Conclusion

Absence of surgical drain placement at the time of kidney transplant had no unfavorable effect on the incidence rate of developing wound complications compared to the use of drains. The limited need-to-do dissection and improved surgical technique appear to be the keys for abandoning drain use even in the presence of higher risk factors for wound complication development.

## Data Availability

The datasets used and/or analyzed during the current study are available from the corresponding author on reasonable request.

## Abbreviations

D: Drain
DF: Drain-free
DGF: Delayed graft function
JP: Jackson Pratt
SE: Standard error
Vs.: Versus
